# Feasibility of nuclear ribosomal region ITS1 over ITS2 in barcoding taxonomically challenging genera of subtribe Cassiinae (Fabaceae)

**DOI:** 10.7717/peerj.2638

**Published:** 2016-12-15

**Authors:** Priyanka Mishra, Amit Kumar, Vereena Rodrigues, Ashutosh K. Shukla, Velusamy Sundaresan

**Affiliations:** 1 Department of Plant Biology & Systematics, CSIR - Central Institute of Medicinal and Aromatics Plants, Research Center, Bangalore, Karnataka, India; 2 Biotechnology Division, CSIR - Central Institute of Medicinal and Aromatic Plants, Lucknow, Uttar Pradesh, India

**Keywords:** Cassiinae, Evolutionary studies, Fabaceae, *In-silico* approach, Phylogenetic signal, Plant DNA barcoding, nrDNA internal transcribed spacer

## Abstract

**Premise of the Study:**

The internal transcribed spacer (ITS) region is situated between 18S and 26S in a polycistronic rRNA precursor transcript. It had been proved to be the most commonly sequenced region across plant species to resolve phylogenetic relationships ranging from shallow to deep taxonomic levels. Despite several taxonomical revisions in Cassiinae, a stable phylogeny remains elusive at the molecular level, particularly concerning the delineation of species in the genera *Cassia, Senna* and *Chamaecrista*. This study addresses the comparative potential of ITS datasets (ITS1, ITS2 and concatenated) in resolving the underlying morphological disparity in the highly complex genera, to assess their discriminatory power as potential barcode candidates in Cassiinae.

**Methodology:**

A combination of experimental data and an in-silico approach based on threshold genetic distances, sequence similarity based and hierarchical tree-based methods was performed to decipher the discriminating power of ITS datasets on 18 different species of Cassiinae complex. Lab-generated **s**equences were compared against those available in the GenBank using BLAST and were aligned through MUSCLE 3.8.31 and analysed in PAUP 4.0 and BEAST1.8 using parsimony ratchet, maximum likelihood and Bayesian inference (BI) methods of gene and species tree reconciliation with bootstrapping. DNA barcoding gap was realized based on the Kimura two-parameter distance model (K2P) in TaxonDNA and MEGA.

**Principal Findings:**

Based on the K2P distance, significant divergences between the inter- and intra-specific genetic distances were observed, while the presence of a DNA barcoding gap was obvious. The ITS1 region efficiently identified 81.63% and 90% of species using TaxonDNA and BI methods, respectively. The PWG-distance method based on simple pairwise matching indicated the significance of ITS1 whereby highest number of variable (210) and informative sites (206) were obtained. The BI tree-based methods outperformed the similarity-based methods producing well-resolved phylogenetic trees with many nodes well supported by bootstrap analyses.

**Conclusion:**

The reticulated phylogenetic hypothesis using the ITS1 region mainly supported the relationship between the species of Cassiinae established by traditional morphological methods. The ITS1 region showed a higher discrimination power and desirable characteristics as compared to ITS2 and ITS1 + 2, thereby concluding to be the locus of choice. Considering the complexity of the group and the underlying biological ambiguities, the results presented here are encouraging for developing DNA barcoding as a useful tool for resolving taxonomical challenges in corroboration with morphological framework.

## Introduction

DNA barcoding is an important tool for research in biodiversity hot-spots based on the identification and standardization of specific region of the plant genome that can be sequenced routinely in diverse sample sets to identify and discriminate species from one another (Hebert et al., 2003; Gregory, 2005). The revolution introduced by DNA barcoding relies on molecularization (variability in molecular markers), computerization (transposition of the data through bioinformatics workbench) and standardization (extension of approach to diverse group) of traditional taxonomical framework to easily associate all life stages of a biological entity (Casiraghi et al., 2010). The short, variable and standardized DNA sequence can be termed as DNA barcode when it mirrors the distributions of intra- and interspecific variabilities separated by a distance called ‘DNA barcoding gap’ and characterizes conserved flanking regions for development of universal primers across highly divergent taxa (Kress et al., 2005; Savolainen et al., 2005; Hollingsworth et al., 2009).

In the past, DNA barcoding in plants has been extensively reviewed (Vijayan & Tsou, 2010; Hollingsworth, Graham & Little, 2011), but still there is a considerable debate on the consensus of the choice of a standard region (Mishra et al., 2016). Apart from the accepted mitochondrial cytochrome oxidase I gene (*COI*) in animals and the nuclear ribosomal internal transcribed spacer (nrITS) region in fungi, the search for an analogous region in plants focused attention on the plastid genome (Chase et al., 2005; Kress et al., 2005; Nilsson et al., 2006; Fazekas et al., 2009). Subsequently, major individual candidate regions *matK*, *rbcL*, *rpoB, rpoC1*, and the intergenic spacers ITS, *trnH-psbA*, *trnL-F*, *atpF-atpH* and *psbK-psbI* were tested for use in plants on their discrimination capacity. Due to pitfalls and challenges associated with a single locus, the combination of loci emerged as a promising choice to obtain appropriate species discrimination (Chase et al., 2007; Kress & Erickson, 2007; Fazekas et al., 2008; CBoL Plant Working Group, 2009; Hollingsworth, Graham & Little, 2011).

The ITS region in plants has been shown to perform as a powerful phylogenetic marker when compared with either coding or noncoding plastid markers due to high copy number of rRNA genes and high degree of variations even between the closely related species (Álvarez & Wendel, 2003; Chase et al., 2007; China Plant BOL Group, 2011; Li et al., 2015). The availability of several universal primer sets and moderate size of 500–750 bp provides an advantageous feature in deciphering the riddles within and among various taxa. The spacer DNA occurs as intercalated in the 16S–5.8S–26S region of rDNA locus and consists of ITS1, ITS2 and the highly conserved 5.8S. Also, many studies have compared the discriminatory power of ITS region in its entirety with ITS2, proposing ITS2 as an alternative barcode to entire ITS region because of sufficient variation in primary sequences and secondary structures (Chen et al., 2010; Gao et al., 2010; Han et al., 2013). Despite the problems in amplifying and directly sequencing the entire region, ITS1 has been tested as a better barcode for eukaryotic species (Wang et al., 2014) and also a successful region for the members of legume family (Yadav et al., 2016).

Fabaceae (Legumes) are the third largest family of flowering plants with Caesalpinioidae being the second largest of the three subfamilies (Irwin & Barneby, 1981). Cassiinae is a subtribe of Fabaceae in the subfamily Caesalpinioidae, comprising of three genera, viz. *Cassia* L. sens. str., *Senna* P. Mill., and *Chamaecrista* Moench. Genus *Cassia* L. sens. *lat*., is one of the twenty-five largest genera of dicotyledonous plant with high diversity of secondary metabolites which serve as medicinal, nutraceuticals and sustainable agriculture etc. (Singh, 2001). Tinnevelly *Senna* is the second largest exported herb drug in the country and contributes significantly in the range of 5,000 metric tons per year as commercial products (Seethapathy et al., 2014). Despite several studies by many taxonomists, either on the whole family or at the genus level, there has been considerable divergence of opinion concerning the delimitations and taxonomic status of the subgenera at the molecular level. The wide variability in habit ranging from tall trees to delicate annual herbs, floral and vegetative features, pods variability, etc had made its taxonomical framework quite complex and intriguing (Singh, 2001). Cytological and karyological studies of 17 taxa of *Cassia*, showed no correlation between the habit and karyotype symmetry of various species (Bir & Kumari, 1982). Thus, the identification of the species has proved tricky and is rather difficult to account for the entire genetic variation existing in the genera. A robust and reliable method is crucial to discriminate plant species to secure their diversity.

Few studies in *Cassia* have been conducted utilizing the dominant molecular markers (Mohanty et al., 2010), plastid and nuclear region markers for different purposes (Purushothaman et al., 2014; Seethapathy et al., 2014). The studies demonstrated the subsequent contribution of markers in assessing product adulteration in herbal drug market in India (Seethapathy et al., 2014). Although the results were not based on evolutionary relationships concept, they did indicate a potential role of different regions (markers) in resolving species complexity in *Cassia* (Mohanty et al., 2010; Purushothaman et al., 2014).

In this study, we evaluated the potential ability of ITS regions for identifying and discriminating subtribe Cassiinae based on a representative sample consisting of approximately half of the genera. The applicability and effectiveness of ITS regions (ITS1 and ITS2) in discriminating species across the genera *Cassia, Senna* and *Chamaecrista* were studied for the first time. The sufficient sequences available in GenBank with nuclear region ITS were included for analysis. The main goals of this study were as follows: (i) to infer applicability and efficacy of the ITS regions (ITS1, ITS2 and ITS1 + 2) as barcoding candidates for subtribe *Cassiinae*; (ii) to test the reliability of the underlying taxonomic monographs at the genome level in resolving congeneric species; and (iii) to compare different methods of evaluating DNA barcodes in these highly complex genera.

## Materials and Methods

### Taxon sampling, DNA amplification and sequencing

A total of 54 accessions of 18 species belonging to three genera viz.* Cassia, Senna,* and *Chamaecrista* from India were examined during the study. For obtaining the sequences generated from molecular experiments in our lab, a total of 18 individuals corresponding to three different genera were collected from different geographical regions of South Western Ghats and Uttar Pradesh. The species were identified and authenticated using the morphological characters described in a monographic study on Cassiinae in India (Singh, 2001) by Dr. V. Sundaresan, Senior Scientist, Central Institute of Medicinal and Aromatic Plants, Research Centre (Bangalore). For each of the species, herbarium specimens were prepared and deposited at the Herbaria of the Central Institute of Medicinal and Aromatic Plants (Table 1, CIMAP Communication No.: CIMAP/PUB/2016/24), Lucknow.

Legumes family produce a high diversity of secondary metabolites, which causes extreme difficulty in isolation of high-quality nucleic acids. Based on literature and commercial kits available, we attempted modification of several previously reported methods to isolate high quality DNA. Ultimately, total genomic DNA from individual accessions was extracted from the leaf tissues (dried in silica-gel) using the modified cetyl trimethyl ammonium bromide (CTAB) protocol with necessary major modifications (Khanuja et al., 1999) and supplementing it with the Nucleospin Plant II Maxi prep kit using the manufacturer’s protocol (MACHEREY-NAGEL, Duren Germany). The concentration of *β*-mercaptoethanol and PVP (Polyvinylpyrrolidone) were increased to 2% v/v and 4% w/v, respectively. An additional chloroform-isoamyl alcohol (96:4) purification step was performed to remove proteins and potentially interfering secondary metabolites. Isolated DNA was checked for its quality and quantity by electrophoresis on a 0.8% agarose gel and spectrophotometric analysis (NanoDrop, ND-1000, USA). The nuclear internal transcribed spacer (ITS1 and ITS2) regions of all the individuals were amplified according to PCR reaction conditions (94 °C, 5 min; (30 cycles: 94 °C, 1 min; 50 °C, 1 min; 72 °C, 1.5 min); 72 °C, 7 min) following guidelines from the CBOL plant-working group and sequenced using universal primers ITS5a forward 5^′^-CCTTATCATTTAGAGGAAGGAG-3^′^ and ITS4 reverse 5^′^-TCCTCCGCTTATTGATATGC-3^′^ (Kress et al., 2005). PCR amplifications for each primer set were carried out in a 50 µl volume solution containing 1x Taq DNA polymerase buffer, 200 µM each dNTPs (dATP:dTTP:dCTP:dGTP in 1:1:1:1 parts), 10 pmol of each primer (forward and reverse), 1 unit of Taq DNA polymerase and ∼25–50 ng of template DNA. The PCR fragment lengths were determined on a 2% agarose gel. The PCR products were purified with Nucleospin PCR purification kit (MACHEREY-NAGEL, Duren, Germany) as per the manufacturer’s instructions. Presence of the specific product was confirmed by running the purified PCR products on 2% agarose gel. All the purified PCR products were subjected to double-stranded sequencing using the Applied Biosystems Big Dye Terminator Cycle Sequencing Kit (Applied Biosystems, Foster City, CA, USA) on an ABI 3130 XL automated sequencer (Applied Biosystems).

**Table 1:** Passport sheet for the samples undertaken. Sample details with GenBank accession numbers of all the samples of *Cassia, Senna, and Chamaecrista* used in this study. Accessions numbers marked in bold represent lab-generated sequences from the present study.

Taxon	Region	Collection site	Voucher number (No.)	GenBank (NCBI) accessions no.
*Chamaecrista absus*	ITS	Tirunelveli, Tamil Nadu	CIMAP-C010	**KT279729.1**
*Chamaecrista absus*	ITS2	GenBank	GenBank	FJ009832.1
*Chamaecrista absus*	ITS	GenBank	GenBank	KC817015.1
*Chamaecrista absus*	ITS2	GenBank	GenBank	FJ009832.1
*Chamaecrista nigricans*	ITS	Tuticorin, Tamil Nadu	CIMAP-C011	**KT279731.1**
*Chamaecrista nigricans*	ITS2	GenBank	GenBank	JQ301845.1
*Chamaecrista nigricans*	ITS2	GenBank	GenBank	JQ301845.1
*Senna uniflora*	ITS	Tirunelveli, Tamil Nadu	CIMAP-C012	**KT279730.1**
*Senna uniflora*	ITS	GenBank	GenBank	KJ605909.1
*Senna uniflora*	ITS	GenBank	GenBank	KJ605897.1
*Senna italica*	ITS	Tuticorin, Tamil Nadu	CIMAP-C013	**KT279732.1**
*Senna italica*	ITS	GenBank	GenBank	KJ004293.1
*Senna italica*	ITS	GenBank	GenBank	KF815503.1
*Senna hirsuta*	ITS	Tirunelveli, Tamil Nadu	CIMAP-C014	**KT279733.1**
*Senna hirsuta*	ITS	GenBank	GenBank	KJ605904.1
*Cassia fistula*	ITS2	GenBank	GenBank	JQ301830.1
*Senna hirsuta*	ITS	GenBank	GenBank	KJ605905.1
*Senna hirsuta*	ITS2	GenBank	GenBank	KJ605904.1
*Senna alata*	ITS	Kukrail, Lucknow	CIMAP-C015	**KT308089.1**
*Senna alata*	ITS	GenBank	GenBank	KJ638414.1
*Senna alata*	ITS	GenBank	GenBank	KJ638413.1
*Senna sulfurea*	ITS	Raebareli, Lucknow	CIMAP-C016	**KT308090.1**
*Senna sulfurea*	ITS2	GenBank	GenBank	JQ301833.1
*Senna siamea*	ITS	CIMAP, Bangalore	CIMAP-C017	**KT308091.1**
*Senna siamea*	ITS	GenBank	GenBank	KC984644.1
*Senna siamea*	ITS	GenBank	GenBank	KJ638421.1
*Senna siamea*	ITS2	GenBank	GenBank	JQ301842.1
*Senna obtusifolia*	ITS	Raebareli, Lucknow	CIMAP-C018	**KT308092.1**
*Senna obtusifolia*	ITS	GenBank	GenBank	GU175319.1
*Senna occidentalis*	ITS	Frlht, Bangalore	CIMAP-C019	**KT308093.1**
*Senna occidentalis*	ITS	GenBank	GenBank	KJ638419.1
*Senna occidentalis*	ITS	GenBank	GenBank	KP092706.1
*Senna occidentalis*	ITS2	GenBank	GenBank	KJ638419.1
*Senna occidentalis*	ITS2	GenBank	GenBank	KP092706.1
*Senna pallida*	ITS	Raebareli, Lucknow	CIMAP-C020	**KT308095.1**
*Cassia fistula*	ITS2	GenBank	GenBank	JQ301830.1
*Senna pallida*	ITS2	GenBank	GenBank	JQ301829.1
*Senna auriculata*	ITS	Frlht, Bangalore	CIMAP-C021	**KT308096.1**
*Senna auriculata*	ITS	GenBank	GenBank	KJ638417.1
*Senna auriculata*	ITS2	GenBank	GenBank	JQ301838.1
*Senna auriculata*	ITS	GenBank	GenBank	KJ638416.1
*Senna alexandrina*	ITS	CIMAP, Lucknow	CIMAP-C022	**KT308097.1**
*Senna alexandrina*	ITS	GenBank	GenBank	KF815491.1
*Senna alexandrina*	ITS2	GenBank	GenBank	JQ301846.1
*Senna alexandrina*	ITS2	GenBank	GenBank	JQ301846.1
*Senna surattensis*	ITS	GenBank	GenBank	KJ638427.1
*Senna surattensis*	ITS	GenBank	GenBank	KJ605903.1
*Senna surattensis*	ITS	GenBank	GenBank	KJ605902.1
*Senna surattensis*	ITS2	GenBank	GenBank	KJ638427.1
*Senna tora*	ITS	GenBank	GenBank	KJ638426.1
*Senna siamea*	ITS2	GenBank	GenBank	JQ301842.1
*Senna tora*	ITS	GenBank	GenBank	KJ638425.1
*Senna tora*	ITS	GenBank	GenBank	KJ638424.1
*Senna tora*	ITS2	GenBank	GenBank	KJ638426.1
*Senna tora*	ITS2	GenBank	GenBank	KJ638425.1
*Senna tora*	ITS2	GenBank	GenBank	KJ638424.1
*Cassia roxburghii*	ITS	GenBank	GenBank	JX856435.1
*Cassia roxburghii*	ITS2	GenBank	GenBank	JQ301841.1
*Cassia javanica*	ITS	Raebareli, Lucknow	CIMAP-C023	**KT338798.1**
*Cassia javanica*	ITS	GenBank	GenBank	FJ009821.1
*Cassia javanica*	ITS2	GenBank	GenBank	JQ301831.1
*Cassia javanica*	ITS	GenBank	GenBank	FJ980413.1
*Cassia javanica*	ITS2	GenBank	GenBank	JQ301831.1
*Cassia fistula*	ITS	SCAD, Tirunelveli	CIMAP-C024	**KT308094.1**
*Cassia fistula*	ITS	GenBank	GenBank	JX856431.1
*Cassia fistula*	ITS	GenBank	GenBank	JX856430.1
*Cassia fistula*	ITS2	GenBank	GenBank	JQ301830.1
*Senna surattensis*	ITS2	GenBank	GenBank	KJ638427.1
*Senna surattensis*	ITS2	GenBank	GenBank	KJ638427.1
*Senna pallida*	ITS2	GenBank	GenBank	JQ301829.1
*Senna auriculata*	ITS2	GenBank	GenBank	JQ301838.1
*Senna auriculata*	ITS2	GenBank	GenBank	JQ301838.1
*Senna hirsuta*	ITS2	GenBank	GenBank	KJ605904.1
*Senna hirsuta*	ITS2	GenBank	GenBank	KJ605904.1
*Senna siamea*	ITS2	GenBank	GenBank	JQ301842.1
*Cassia javanica*	ITS2	GenBank	GenBank	JQ301831.1
*Cassia javanica*	ITS	GenBank	GenBank	FJ009821.1
*Cassia roxburghii*	ITS	GenBank	GenBank	JX856435.1
*Cassia roxburghii*	ITS2	GenBank	GenBank	JQ301841.1

Apart from the lab-generated sequences, all the nucleotide sequences belonging to genera *Cassia, Senna,* and *Chamaecrista* for the regions ITS1 and ITS2 were downloaded from the NCBI based on the BLAST results. The sequences were filtered on the basis of length (less than 300 bp were omitted), lack of voucher specimens as well as verification (sequences categorised as unverified in GenBank were omitted). An effort was made to include minimum five individuals for each species, but due to unavailability of sequences for few species in the NCBI database and difficulty in obtaining the species in the field, the representatives of each species were limited to three. The GenBank accession numbers used in this study are listed in Table 1.

### Data analysis

Electropherograms corresponding to raw sequences of individual accessions from both the forward and reverse primers were assembled and edited using CodonCode Aligner v.3.0.1 (CodonCode Corporation). Sequences were clipped at the end to avoid the presence of variable sites introduced by the sequencing artefacts. Due to its well-conserved nature, the 5.8S gene region was removed from any sequence so that the ITS1 and ITS2 regions could be analyzed separately and concatenated. The edited sequences were then aligned with MUSCLE 3.8.31 on the EMBLEBI website (http://www.ebi.ac.uk) with default parameter and adjusted manually in BioEdit v7.1.3.0 (Hall, 1999). All the variable sites were rechecked on the original trace files. To evaluate the effectiveness of ITS1, ITS2 and their combination (ITS1 + 2) as barcodes in the concerned genera, three widely used methods viz. distance-based (PWG-distance), similarity-based and tree-based were applied.

### Genetic distance-based method

To evaluate the measure of effective barcode locus, DNA barcoding gap was calculated using TaxonDNA software with a ‘pairwise summary’ function under K2P nucleotide substitution model (Meier et al., 2006). The pairwise genetic distance were calculated at the observed levels of intra- and inter-specific divergence for each barcode. To test the accurate species assignments, the distributions of the pairwise intra- and inter-specific distances with 0.005 distance intervals were generated. The histogram of distances vs. abundance were plotted to estimate the presence of any barcoding gaps. For the PWG-distance method, the genetic pairwise distance was estimated by MEGA version 6 (Tamura et al., 2013) using the Kimura two-parameter distance model (K2P) with pairwise deletion of missing sites (Kimura, 1980). Average inter-specific distance was used to characterize inter-specific divergence (Meyer & Paulay, 2005; Meier, Zhang & Ali, 2008) and ‘all’ intra-specific distance, mean ‘theta’ and coalescent depth were used to characterize intra-specific distances. Finally, the obtained inter- and intra-specific distances were plotted with frequency distribution in bin interval of 0.05 to illustrate the existing DNA barcoding gap (Meyer & Paulay, 2005; Lahaye et al., 2008).

### DNA sequence similarity-based method

To test the potentiality of ITS regions to identify species accurately based on sequence similarity, the proportion of correct identifications were calculated using SpeciesIdentifier program from the TAXONDNA software package with ‘Best match’ (BM), ‘Best close match’ (BCM) and ‘All species barcodes’ functions (Meyer & Paulay, 2005). The tool examines all the sequences present in aligned data set and compares each successive sequence with all the other sequences to determine the closest match. The ‘Best match’ modules than classifies the sequences as correct and incorrect based on the indicated pair from the similar species or different species respectively. While the various equally best matches from different species are referred to be as ambiguous. The ‘Best close match’ module works on the intra-species variability criterion and considered to be the more rigorous method in TaxonDNA. The sequences classified as ‘no match’ are the results above the calculated threshold value (Meier et al., 2006).

### Tree-based method

To evaluate the ability of candidate barcode to delimit the species into discrete clades or monophyletic groups, three different optimality criteria (tree-building method) viz Neighbour-joining with minimum evolution (NJ), maximum likelihood (ML) and Bayesian inference (BI) were employed. To test the reliability of the result, NJ and ML trees were constructed and compared with two different softwares: (i) In MEGA using the K2P distance as model of substitution (Tamura et al., 2013) and (ii) In PAUP 4.0 with the HKY-gamma substitution model (Swofford, 2003). The reliability of the node was assessed by a bootstrap test with 1,000 pseudo-replicates with the K2P distance options (Felsenstein, 1988). Bayesian sampling was performed in BEAST1.8 using the operators: HKY substitution model with four gamma categories, a constant-rate Yule tree prior and 10,000 chain lengths and all other priors and operators with the default settings. Coalescent tree priors were used for population-level analysis and speciation prior were applied to estimate relationships and divergence times of inter-species data. Trees were sampled for every 5,000 generations resulting in a total of 10,000 trees, and a burn-in of 50,00,000. Beast file was created using the BEAUti program v1.8.2 within Beast and performance of each run was further analysed with the program Tracer (Rambaut et al., 2014). The resulting Beast tree files were annotated through TreeAnnotator v1.8.2 and visualized and edited with FigTree v1.4.2. (Rambaut, 2014, http://tree.bio.ed.ac.uk/software/figtree). Visualization and analysis of all the resulting trees through PAUP 4.0 was done in Dendroscope3 (Huson & Scornavacca, 2012). Gaps were treated as missing data for all the phylogenetic analysis.

## Results

### PCR amplification and sequence characteristics

The sequence characteristics of ITS regions evaluated in this study showed good success rates (90%) for PCR amplification (ranging from 571-1153 bp with mean size ∼707 bp; gel images can bé provided on request) and sequencing in both the direction using a single primer pair ITS5a forward and ITS4 reverse. The presence of large amount of secondary metabolites, polysaccharides and polyphenolic compounds in the plants of sub-family Caesalpinioidae, hindered the isolation of pure nucleic acids. Therefore few samples had to be excluded from the study after 3–4 initial amplification attempts that failed due to the presence of inhibitory components. The present study generated 15 new sequences belonging to 15 different species of *Cassia, Senna,* and *Chamaecrista.* The sequences were submitted to NCBI (www.ncbi.nlm.nih.gov/genbank/) and corresponding GenBank accession numbers were obtained for each species. A total of 64 sequences corresponding to 18 different species of *Cassia, Senna*, and *Chamaecrista* for ITS regions (ITS1 and ITS2) were obtained from NCBI and included in the study (Table 1). The ITS1 region had an aligned length of 315 bp (Alignment S1) which was greater than that of ITS2 with 258 bp (Table 2; Alignment S2). The combined region ITS1+2 showed an align length of 573 bp (Alignment S3) with 80.1 % of pairwise identity (Table 2). The aligned ITS1 matrix consisted of 315 bp with 206 parsimony sites. The number of variable sites was 210. The maximum intra-specific divergence was observed among the individuals of *Senna siamea* with 0.023 PWG-distance while minimum inter-specific distances were recorded between *Senna hirsuta* and *Senna occidentalis* with 0.039 PWG-distance. The species of genus *Chamaecrista* showed lowest K2P distances (Table 3). Overall the summary statistics for DNA alignments and DNA sequences for the ITS dataset evaluated in this study are summarized in Tables 2 and 3 respectively.

**Table 2:** Summary statistics for DNA alignments.

Alignments	Region	Residual length	G +**C (%)**	Identical sites (%)	Pairwise identity (%)
Alignment S1	ITS1	315	57.0 %	26.3 %	82.15 %
Alignment S2	ITS2	258	63.9 %	35.8 %	77.20 %
Alignment S1+S2	ITS1 + 2	573	60.1 %	30.8 %	80.10 %

**Notes.**

Residual length, the length of the complete alignment, counting portions excluded from analysis; G + C, the G + C content of the complete (total length) alignment; Identical sites, the % of columns in the alignment for which all sequences are identical; Pairwise identity, the % of pairwise residues that are identical in the alignments, including gap versus non-gap residues, but excluding gap vs. gap residues.

**Table 3:** Summary of sequence characteristics of the barcode candidates and their combinations analysed in this study.

Characters	ITS1	ITS2	ITS1 + 2
Aligned length (bp)	315	258	573
Average intra-distance	0.01%	0.03%	0.01%
Average inter-distance	0.24%	0.25%	0.17%
Average theta (e)	0.27%	0.26%	0.18%
Coalescent depth	0.02%	0.38%	0.17%
Proportion of variable sites	66.66%	60.24%	46.53%
Proportion of parsimony sites	65.39%	47.54%	43.64%

### Genetic divergence and Barcoding gap

**Figure 1:**
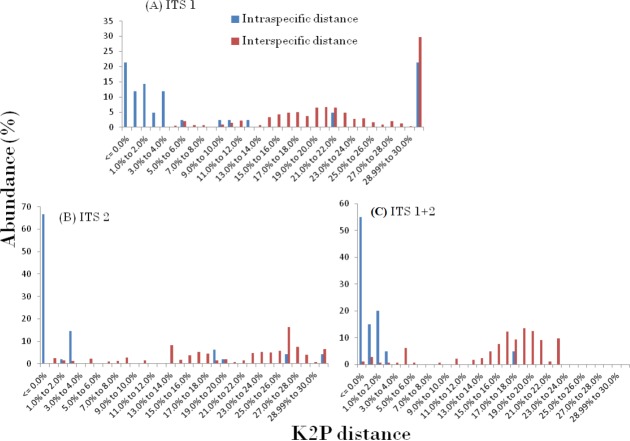
Pairwise distance based on K2P method. Relative abundance of intra- and inter-specific Kimura-2-Parameter pairwise distance based on TaxonDNA methods considering nrITS dataset in genera *Cassia, Senna*, and *Chamaecrista*.

The presence of DNA barcoding gap based on the concept of an inter-specific distance being larger than the intra-specific distance for a species, directly reveals the species discrimination ability of candidate barcodes. In this study, the relative distribution of frequencies of K2P distances for three ITS datasets using TaxonDNA software showed a significant pattern with the inter-specific distance being higher and did not fully overlap with the intra-specific distance resulting in the presence of an identified barcoding gap in the genera. The observed pattern of ITS1, ITS2 and ITS1 + 2 results are presented in Fig. 1. The mean intra- and inter-specific genetic divergence based on PWG distances through MEGA, for ITS1 varied in the range from 0.023 to 0.000 and 0.033–1.185 respectively (Table 3).

### Species discrimination based on different analytical methods

**Figure 2:**
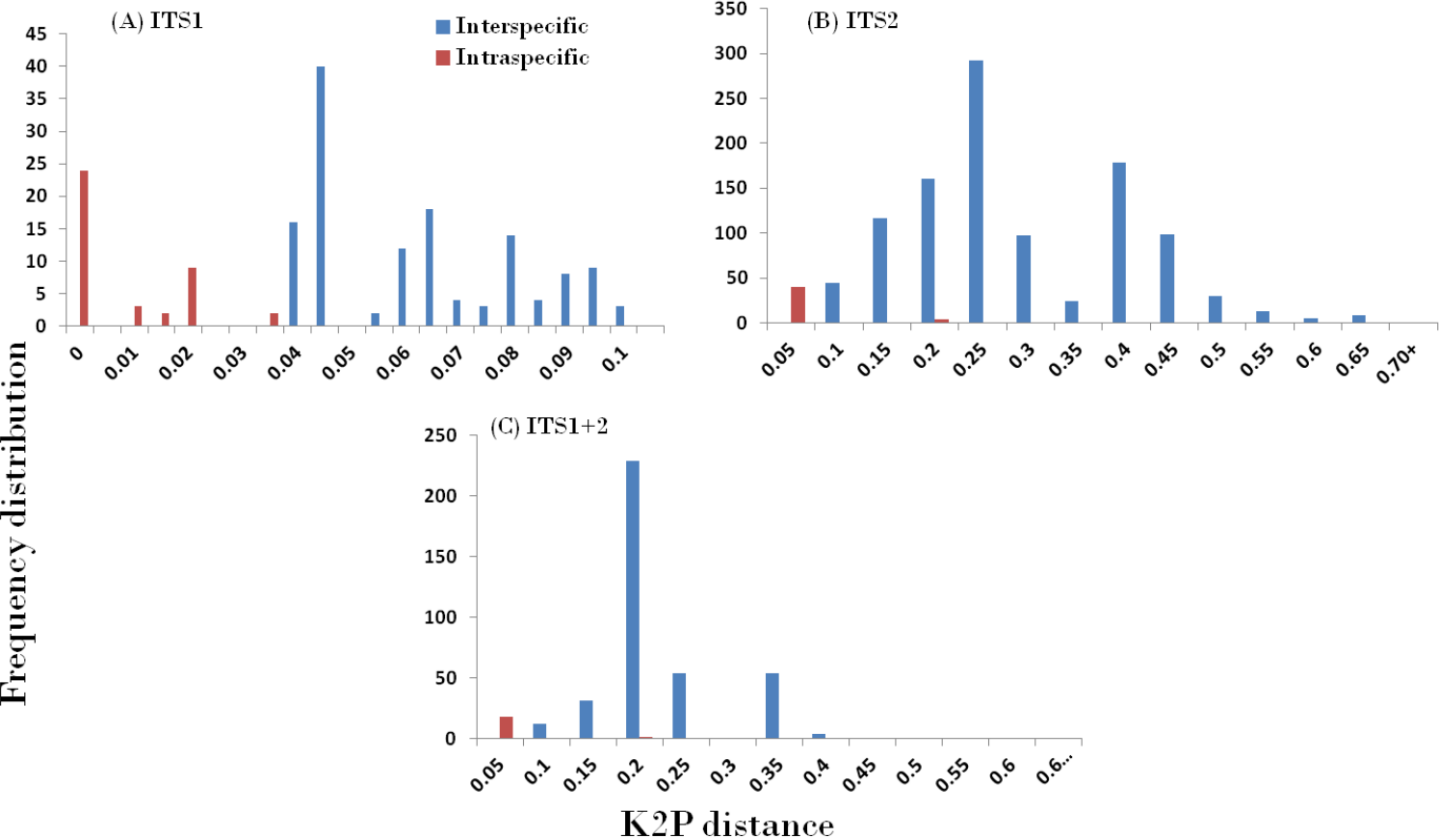
Evaluation of DNA barcoding Gap. Relative distributions of intra- and inter-specific distances based on PWG-distance based methods for the three nrITS datasets in Cassiinae. *x* axes relate to Kimura 2-parameter (K2P) distances arranged in intervals, and the *y* axes correspond to the frequency distribution.

In accordance with the CBOL PWG-distance method, a favourable barcode should possess a high inter-specific divergence to distinguish different species. The result obtained through the different datasets showed significant pattern of inter-specific divergence, whereby ITS1 was concluded to be the best among the candidates. The mean pairwise inter-specific distances were found to be higher in comparison to intra-specific distances in all the barcodes, resulting in the presence of a clear barcode gap. The distance distribution range of all inter- and intra-specific distances for all markers are shown in Fig. 2.

Compared with the PWG- distance method, the BM and BCM functions of TaxonDNA showed the better discrimination success. All the three datasets presented same success rate of species identification when BM was selected in comparison to BCM. The highest and same rate of discriminatory power (81.6%) was observed for ITS1 on both BM and BCM functions. The other two datasets; ITS2 and ITS1 + 2 datasets recovered 75.0% and 77.4% BM respectively (Table 4).

**Table 4:** Identification success rates based on analysis of the ‘Best match,’ ‘Best close match’ and ‘All species barcodes’ function of TaxonDNA software for each ITS dataset.

Region	Best match			Best close match			All species barcodes		
	Correct (%)	Ambiguous (%)	Incorrect (%)	Correct (%)	Ambiguous (%)	Incorrect (%)	Correct (%)	Ambiguous (%)	Incorrect (%)
ITS1	81.63	8.16	10.2	81.63	8.16	10.2	30.61	63.26	6.12
ITS2	75.0	0	25.0	75.0	0	25.0	33.33	62.5	4.16
ITS1 + 2	77.41	19.35	3.22	77.41	19.35	3.22	19.35	77.41	3.22

**Figure 3:**
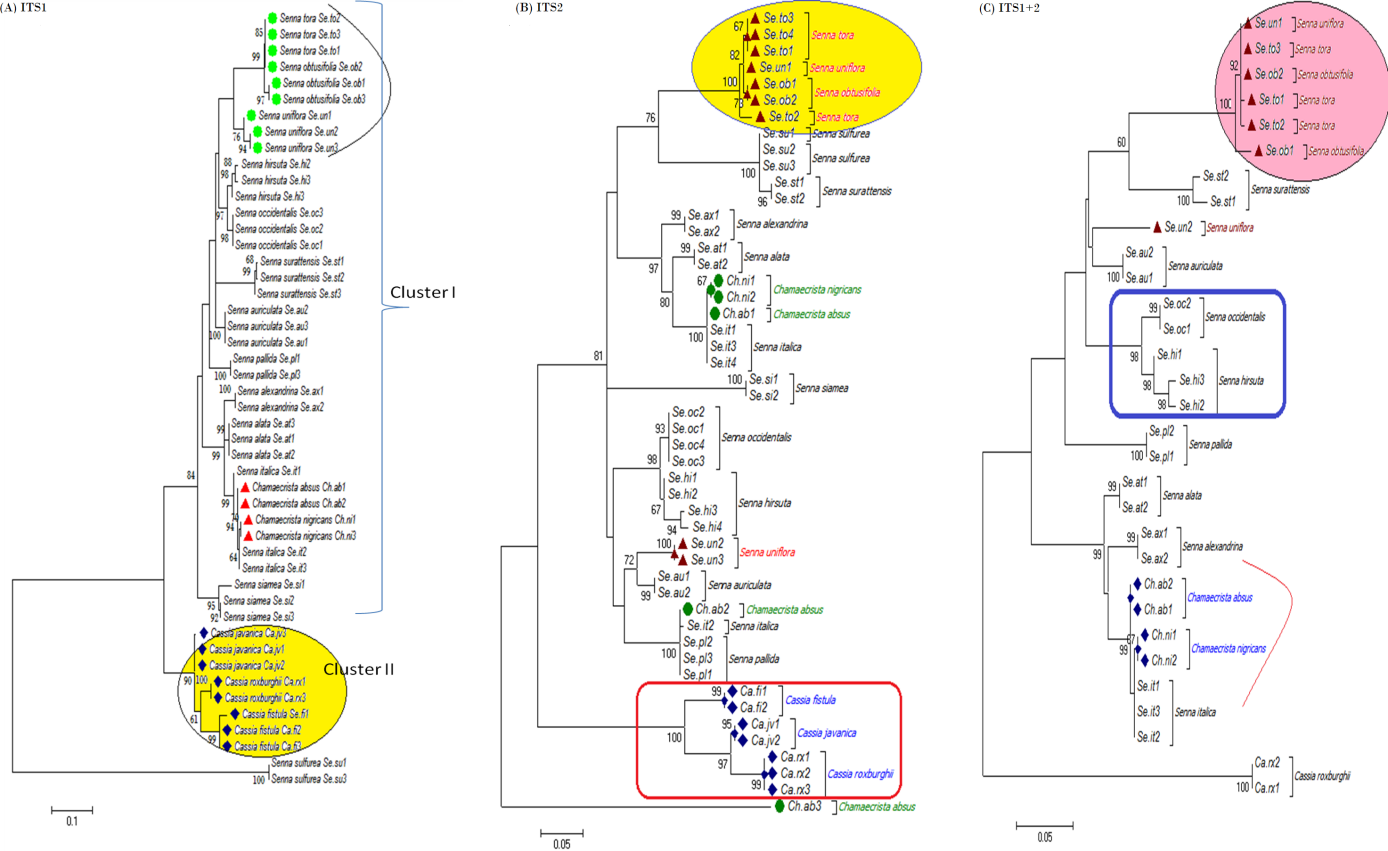
Phylogenetic consensus tree constructed using bayesian inference algorithm. Phylogenetic consensus tree obtained for *Cassia*, *Senna*, and *Chamaecrista* species based on nrITS datasets constructed using the Bayesian inference algorithm. Representatives from individual species are abbreviated based on corresponding taxon.

**Figure 4:**
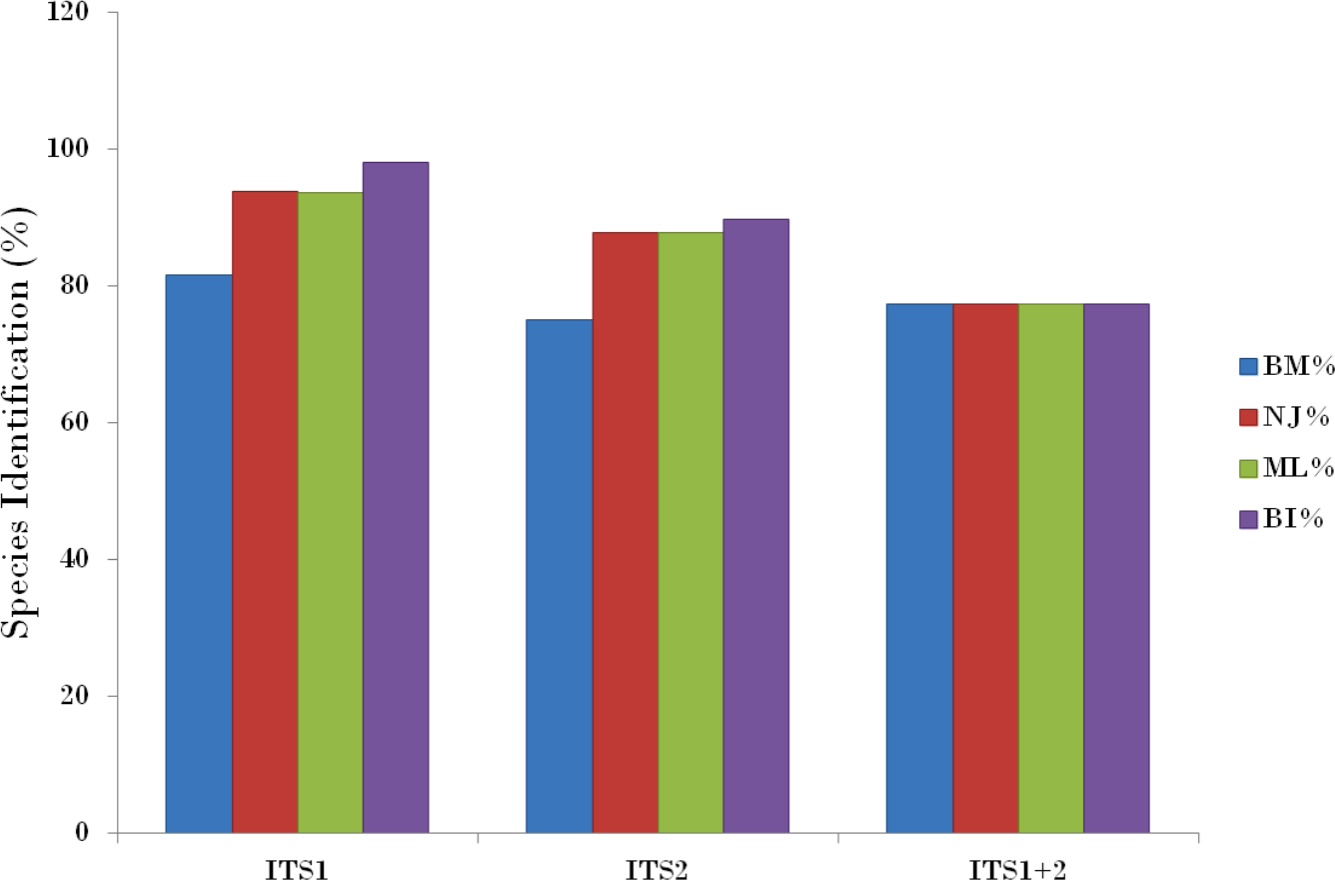
Comparison of species discrimination rates. Species discrimination rates of nrITS datasets based on different methods in Cassiinae. ITS1 barcode in conjunction with the Bayesian inference analysis of hierarchical tree-based method met the objectives of DNA barcoding.

**Figure 5:**
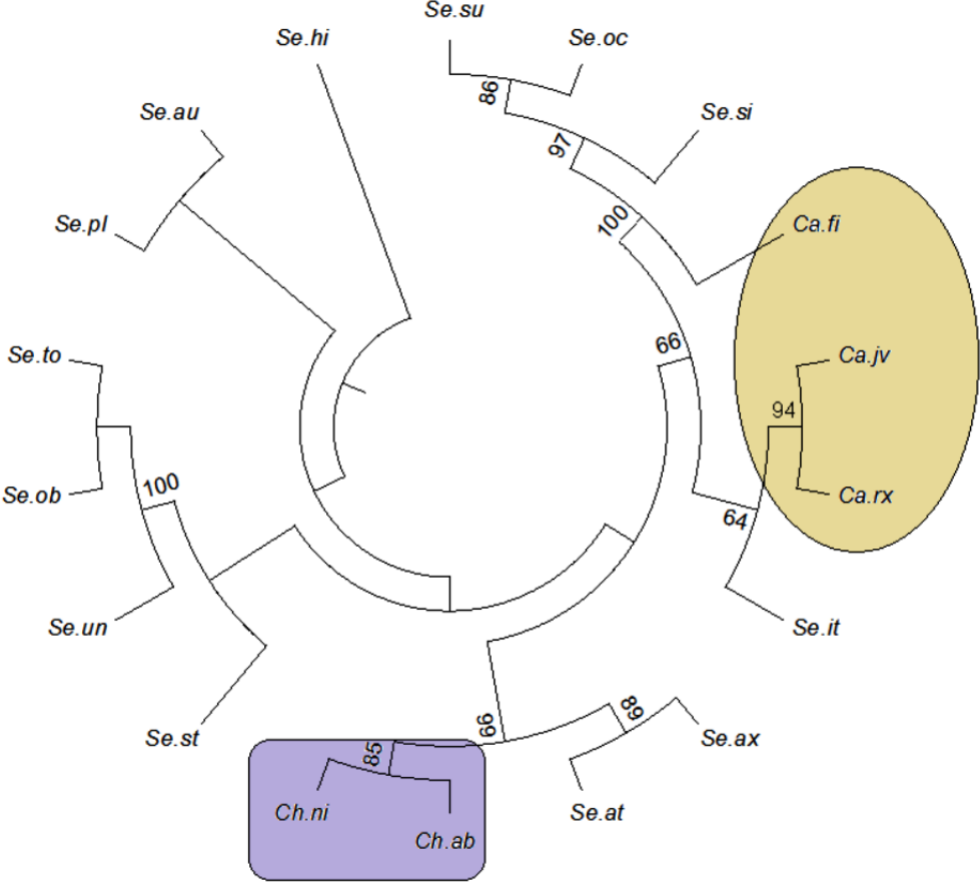
Evolutionary relationships in Cassinae. Evolutionary relationships in genera *Cassia*, *Senna,* and *Chamaecrista* based on nrITS barcode constructed using the Bayesian inference algorithm. Taxon names are abbreviated (see Table 1).

The tree building methods for the evaluation of barcode sequences were estimated based on the correct assignment of individuals forming a monophyletic clade (Fig. 3 and Fig. S1). Among the different phylogenetic methods, BI recovered the highest value for species monophyly in all the datasets. While in the combination of ITS1 + 2, all the three methods viz. NJ, ML and BI provided near similar topology, concluding 77.41% of individuals identified correctly (Fig. 4). The resulting bootstrap value lends support to our findings. Comparing the potentiality of the ITS datasets and the phylogenetic algorithms employed, the highest discriminatory power was observed when ITS1 was used alone, which successfully maintained the genera (*Cassia, Senna,* and *Chamaecrista*) monophyly with few exceptions (Fig. 5). The coalescent and speciation tree priors intrinsically correlated the rate of evolution and time in inferring genetic differences between species. It is interesting to conclude that all the species from genera *Senna* and* Cassia* framed in two different clusters viz. Cluster I and II according to traditional morphology. The phylogenetic tree presented a slight divergence in the clustering of *Chamaecrista absus* accession obtained from GenBank which might be due to the mis-identification of samples. Referring to the species relationships within genera; to some extent, the phylogenetic relationships obtained were in consistent with the result obtained from the traditional morphological classification method. The clustering pattern of three different genera *Cassia, Senna,* and *Chamaecrista* within the subtribe Cassinae based on the nuclear ribosomal region ITS1, proved to be successful in comparison to the infrageneric clustering of taxa. The clustering of *Senna tora*, *Senna uniflora* and *Senna obtusifolia* accessions based on molecular algorithm of ITS1 complies with the morphological similarity occurs among them, while in ITS2, *Senna uniflora* showed little divergence (Fig. 3). Also, we were not able to find out the clear pattern of lineage of respective species within the genus at a molecular level, as according to traditional taxonomy. Worthy to note here, that the resulting pattern within the individuals of same species and high reliability value obtained for their nodes concludes the existence of genetic similarity among them. Framing of *Senna occidentalis* and *Senna hirsuta* into the individual cluster through ITS1, were in consistent with the key classification (Fig. 3).

Besides, all the tree species belonging to genus *Cassia*, undertaken in this study framed an individual cluster (Cluster II) according to their diversity there by concluding the importance of molecular characterization in corroboration with morphological methods in biosystematics study. The analysis conducted in subtribe Cassiinae with the tree based, similarity based and distance based methods showed that BI phylogenetic method and BM similarity methods outperformed the PWG- distance method when using these barcode loci (Fig. 4).

## Discussion

### Discrimination success

Hitherto several different analytical methods were framed for the assessment of the species discrimination ability, which includes tree-based (NJ, MP, Bayesian), distance-based (PWG-distance, p-distance, K2P-distance) and sequence similarity-based methods (BLAST and TaxonDNA), etc., and all of them show different discrimination power on the same data set (Little & Stevenson, 2007; Austerlitz et al., 2009; China Plant BOL Group, 2011; Sandionigi et al., 2012). In this study, sequence analysis of ITS datasets using Bayesian inference (BI) tree-based method gave the highest species resolution based on the topology with the highest product of posterior clade probabilities across all nodes followed by BM and BCM model of TaxonDNA, which too presented equally efficient results either in single or combination of barcodes. Similarly, patterned results have been obtained in different DNA barcoding studies in various plant groups (Yan et al., 2014; Giudicelli, Mäder & De, 2015; Xu et al., 2015; Yan et al., 2015). The clustering algorithm of the Bayesian framework provides a flexible way to model rate variation and obtain reliable estimates of speciation times, provided the assumptions of the models be adequate (Drummond et al., 2012).

The PWG-distance method based on simple pairwise matching recommended by CBOL Plant Working Group as a universal and robust method for the assessment of clear barcoding gap indicated the significance of ITS1, thereby highest number of variable and informative sites (210 and 206, respectively) were obtained. Moreover, the rate of species discrimination is equally efficient when ITS1 and ITS2 are concatenated. These results were expected, considering the complexity of the genera and directly reflected on the performance of ITS1 and ITS2 as barcode markers in *Cassia, Senna,* and *Chamaecrista*. The possible reason behind the results might be the inter-specific sharing of identical sequences or failure of conspecific individuals to group together. Besides, many other aspects have also been reported for unclear barcoding gap such as imperfect taxonomy, inter-specific hybridization, paralogy and incomplete lineage sorting (Yan et al., 2015). However, ITS region has proved to be a suitable marker in authentication of *Cassia* species in the commercial herbal market (Seethapathy et al., 2014). The strong identification ability of nuclear region ITS have been verified in many complex groups (Baldwin et al., 1995; Alves et al., 2014; Wang et al., 2014; Giudicelli, Mäder & De, 2015). Therefore, we suggest that ITS1 itself could be the first option for DNA barcoding in subtribe Cassiinae, though ITS2 should not be discarded.

Moreover, the differences among the three methods compared here, have their possible cause in the theories behind their algorithms and the matter of comprehensive sampling. Thus the comparison of species resolution between studies without consideration of the methods should be avoided for one or the other reasons discussed, as species resolution is an important criterion for assessment of robust barcodes.

### Biological implications of ITS based signalling in Cassiinae

The corroboration of morphological, ecological, geographical, reproductive biology and DNA sequence information paved the successful path for constructing robust taxonomy for diverged plant taxa (DeSalle, Egan & Siddall, 2005; Fazekas et al., 2009; Hollingsworth, Graham & Little, 2011). The ITS region appears to evolve more rapidly than coding regions in interpreting phylogenetic relationships at lower taxonomic levels (Inter-generic and Inter-specific). Species discrimination for the genera *Cassia, Senna* and *Chamaecrista* sampled in this study was high with the strong identification ability of nuclear region ITS. All the three genera maintained the monophyly of the clade either alone or in combination of barcoded loci. The resulting bootstrap value lends support to our findings. To some extent, the divergence of species within the genus did not outperformed as designated according to key taxonomy. The possible reasons behind the findings could be the complexity of the genus with large number of highly polymorphic species which has been found to devise greater interspecific variation (Mohanty et al., 2010). Sometimes interspecific hybridization and gene introgression had accounted for the limited barcoding event at genus level. Moreover genera *Cassia* and *Senna* accounts for high morphological complexity based on species polymorphism, which have been reported in few studies in the past. Successful PCR amplifications, sequencing strategy and alignment matrix obtained from the present study provided further evidence to support the separation of species and genera. The robust phylogenetic signalling of ITS region seems obvious in Cassinae. Although an earlier study (excluding ITS) did not report any single novel region to differentiate the existing *Cassia* species (Purushothaman et al., 2014), our findings provide the potentiality of the ITS region with data support. The delineation of genera based on ITS regions provided a basic framework to have an authentication prospect of correct species at the industrial level.

## Conclusions

Our results show that ITS1 and ITS2 present all the desired characteristics of a DNA barcode for the Cassiinae group examined in the present study. The high rate of PCR amplification and sequencing success coupled with a potentially high rate of correctly assigned species among the genera *Cassia, Senna,* and *Chamaecrista* conclude the discriminating capability of the nuclear region ITS. However, till date, there has been much controversy over the ideal barcode for plants. The previously advocated plastids regions have been used successfully in many barcoding studies (Kress & Erickson, 2007; CBoL Plant Working Group, 2009). In many cases, the potentiality of species discrimination based on the combination of ITS and plastid loci or ITS2 alone has been demonstrated in different plant groups (Pang et al., 2010; Yang et al., 2012; Han et al., 2013; Zhang, Duan & Zhou, 2014). The choice of ITS1 over ITS2, have been suggested recently in the studied taxonomic group (Wang et al., 2014). Through our study, we concluded that ITS1 region should be used as a starting point to assign correct identification in the highly complex genera *Cassia, Senna* and *Chamaecrista.*

##  Supplemental Information

10.7717/peerj.2638/supp-1 Figure S1 Phylogenetic consensus tree obtained using maximum likelihood algorithm Phylogenetic consensus tree obtained for *Cassia*, *Senna,* and *Chamaecrista* species based on nrITS datasets constructed using maximum likelihood algorithm.

10.7717/peerj.2638/supp-2 Supplemental Information 1 The aligned sequences matrix of ITS1

10.7717/peerj.2638/supp-3 Supplemental Information 2 The aligned sequences matrix of ITS2

10.7717/peerj.2638/supp-4 Supplemental Information 3 Concatenated aligned sequences matrix of ITS1+2

## Additional Information and Declarations

### DNA Deposition

The following information was supplied regarding the deposition of DNA sequences: GenBank database.
KT279729; KT279730; KT279731; KT279732; KT279733; KT308089; KT308090; KT308091; KT308092; KT308093; KT338798; KT308094; KT308095; KT308096; KT308097.

### Data Availability

The following information was supplied regarding data availability: The raw data has been supplied as Supplemental File.
