# Supplementary figures and images for: Feasibility of nuclear ribosomal region ITS1 over ITS2 in barcoding taxonomically challenging genera of subtribe Cassiinae (Fabaceae)
Source: PeerJ. 2016 Dec 15;4:e2638. doi: 10.7717/peerj.2638 (PMC5162394; doi:10.7717/peerj.2638)

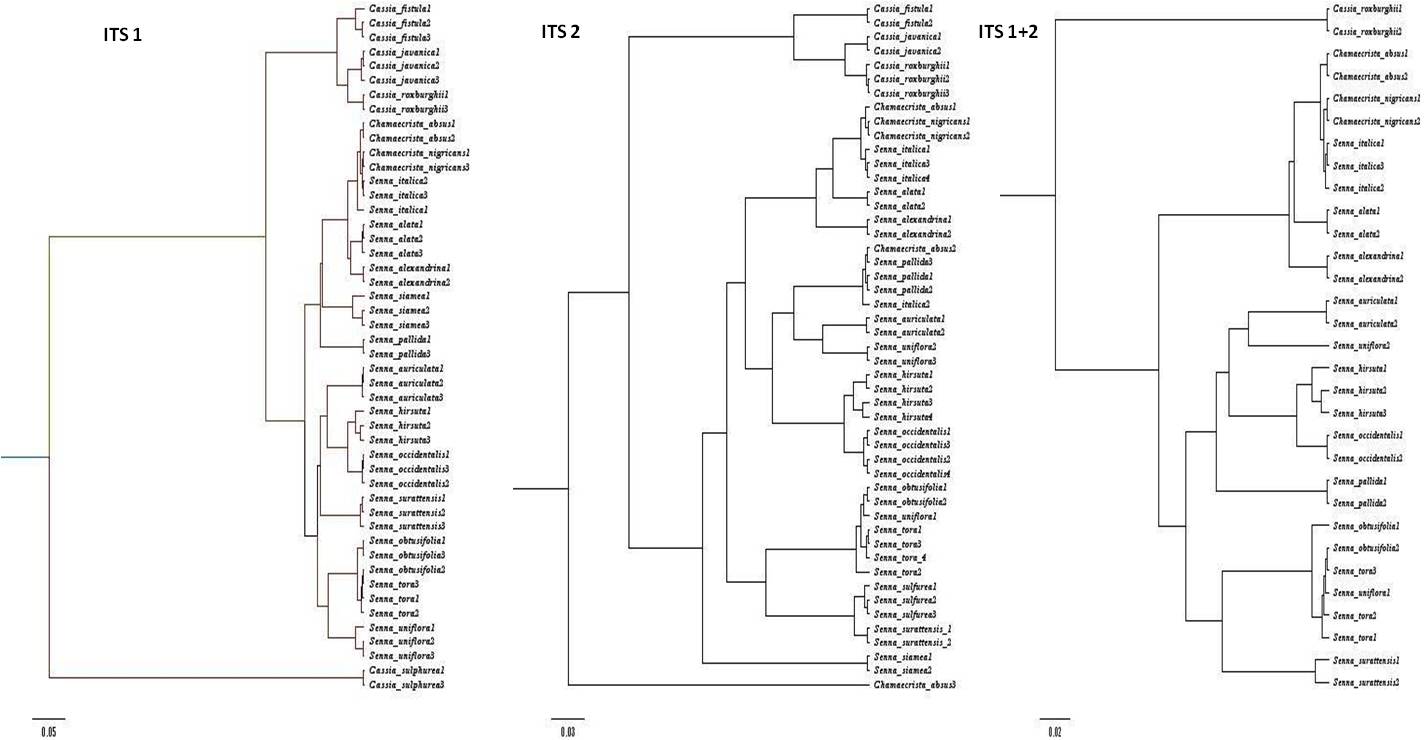

Supplement: Figure S1 — Phylogenetic consensus tree obtained for Cassia, Senna, and Chamaecrista species based on nrITS datasets constructed using maximum likelihood algorithm. [file peerj-04-2638-s001.jpg]
